# Supplementary figures and images for: Transposable Elements Shape the Genome Diversity and the Evolution of Noctuidae Species
Source: Genes (Basel). 2023 Jun 10;14(6):1244. doi: 10.3390/genes14061244 (PMC10298559; doi:10.3390/genes14061244)

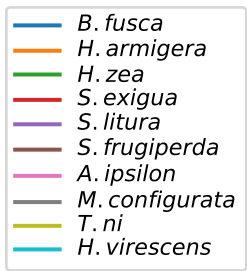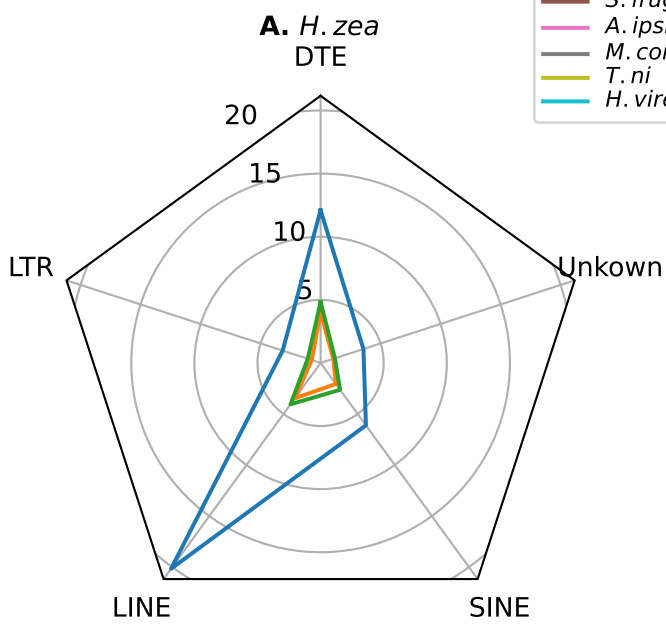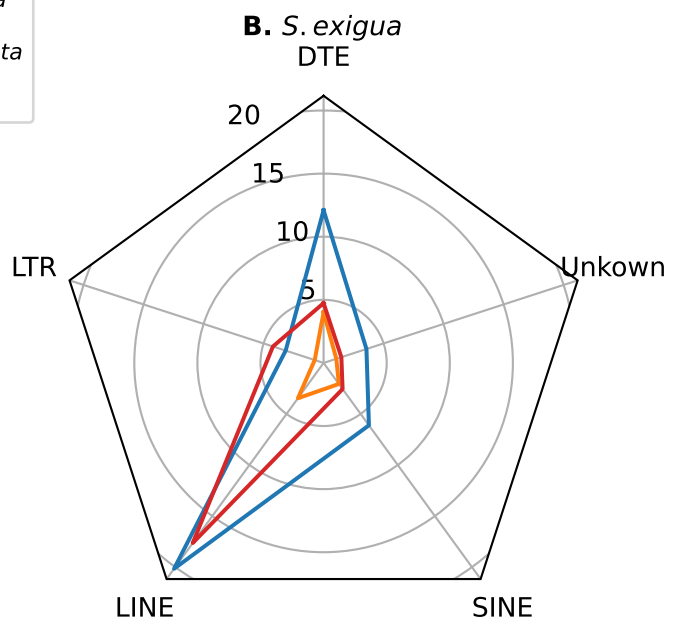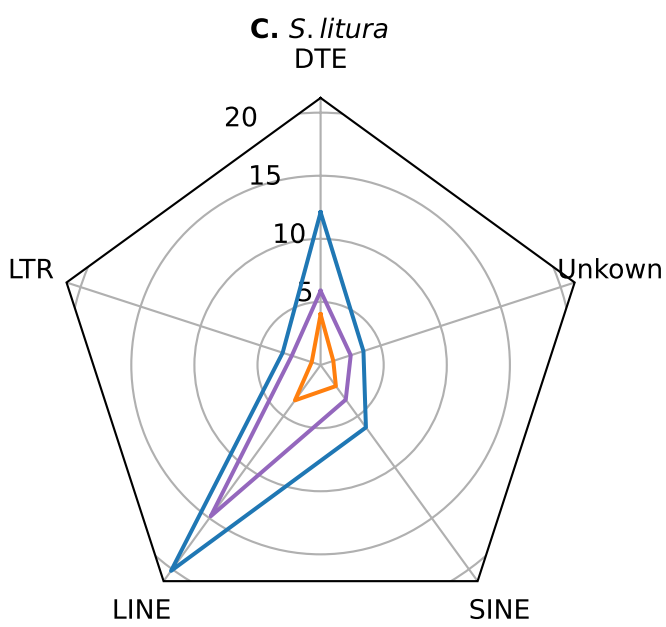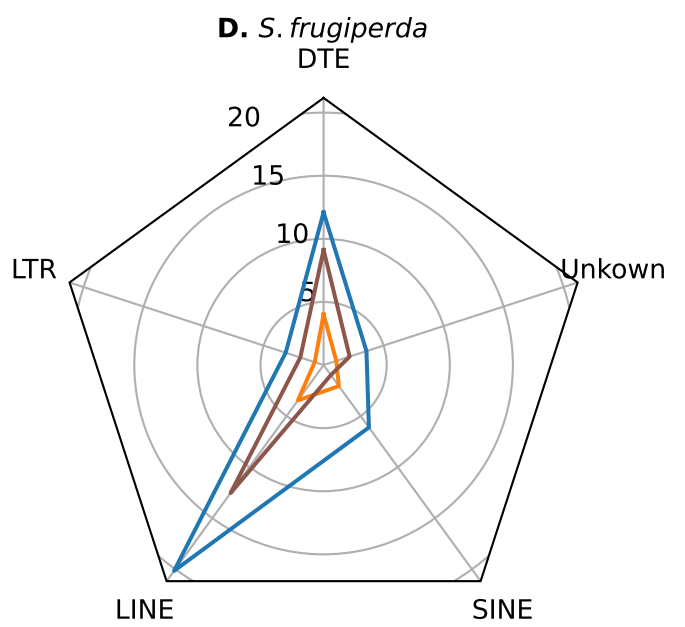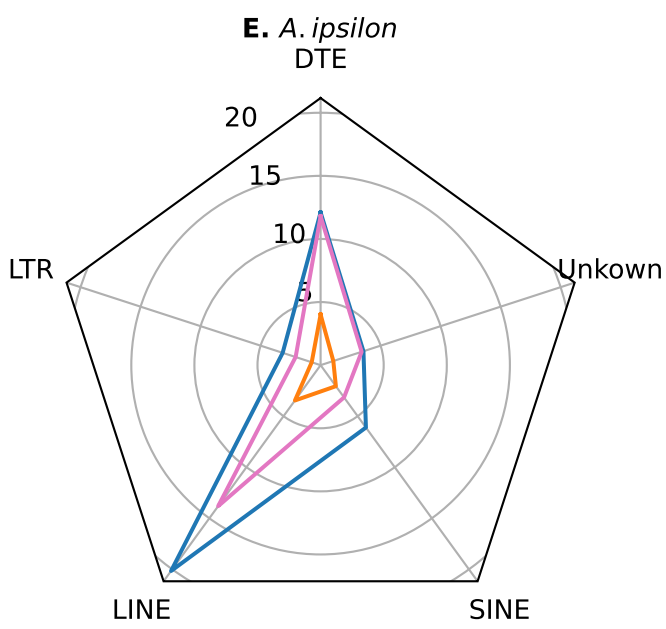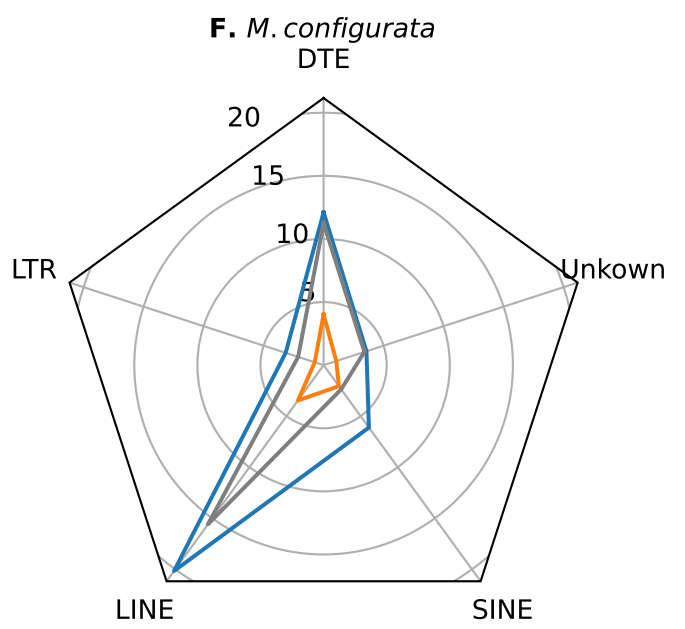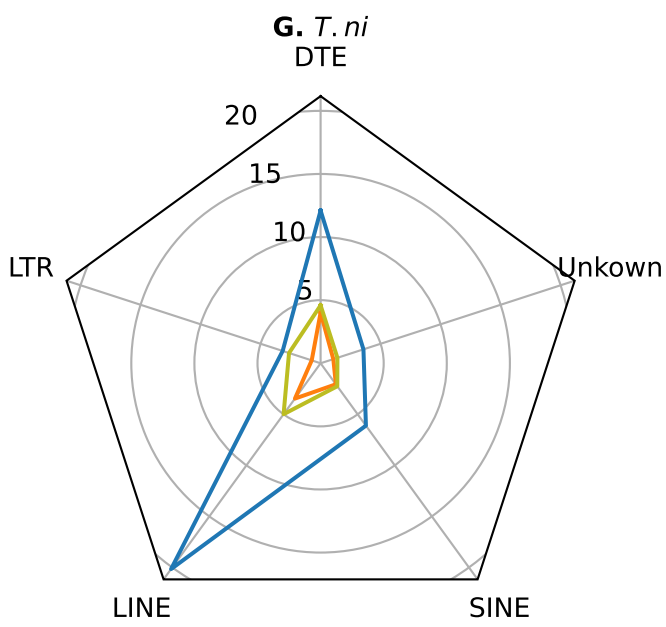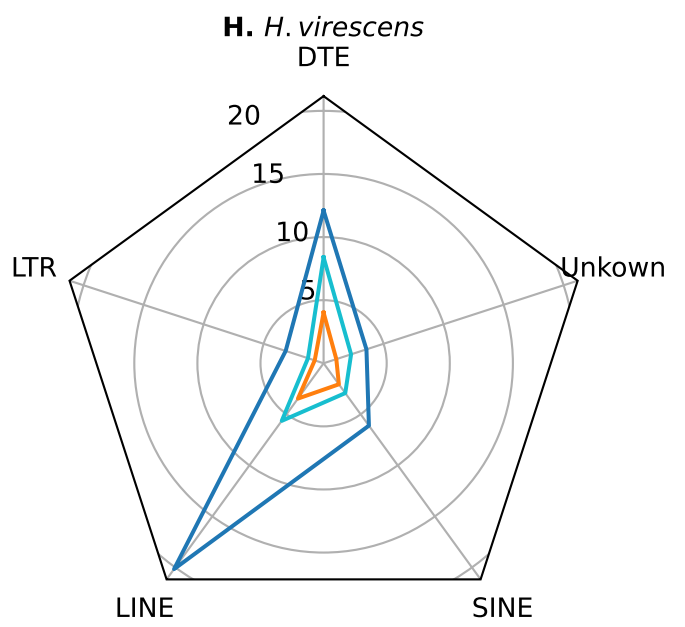

Supplement: Supplementary file 1 [file genes-14-01244-s001.zip › Figure/Figure S1.pdf]

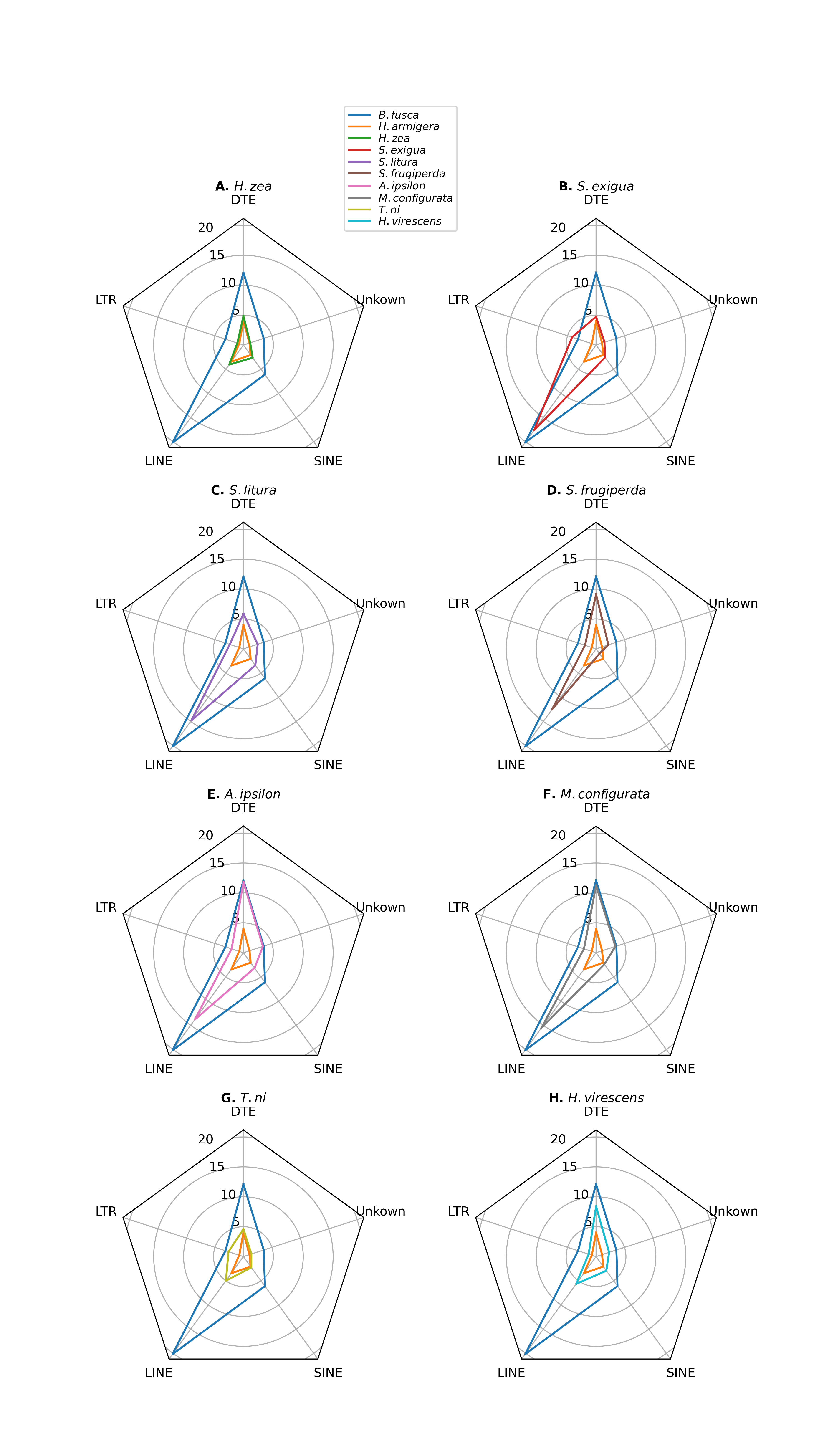

Supplement: Supplementary file 1 [file genes-14-01244-s001.zip › Figure/Figure S1.png]
